# Smoking Protective and Risk Factors Among Transgender and Gender-Expansive Individuals (Project SPRING): Qualitative Study Using Digital Photovoice

**DOI:** 10.2196/27417

**Published:** 2021-10-06

**Authors:** Andy SL Tan, Priscilla K Gazarian, Sabreen Darwish, Elaine Hanby, Bethany C Farnham, Faith A Koroma-Coker, Jennifer Potter, Suha Ballout

**Affiliations:** 1 Annenberg School for Communication University of Pennsylvania Philadelphia, PA United States; 2 Leonard Davis Institute of Health Economics University of Pennsylvania Philadelphia, PA United States; 3 College of Nursing and Health Sciences University of Massachusetts Boston Boston, MA United States; 4 Department of Medicine Harvard Medical School Boston, MA United States; 5 The Fenway Institute Boston, MA United States; 6 Division of General Internal Medicine Beth Israel Lahey Health Boston, MA United States

**Keywords:** transgender and gender expansive populations, tobacco-related health disparities, United States

## Abstract

**Background:**

Transgender and gender-expansive (TGE) adults are twice as likely to smoke cigarettes than cisgender individuals. There is a critical gap in research on effective and culturally sensitive approaches to reduce smoking prevalence among TGE adults.

**Objective:**

This study aims to qualitatively examine the risk and protective factors of cigarette smoking among TGE adults through real-world exemplars.

**Methods:**

We conducted a digital photovoice study among a purposeful sample of 47 TGE adults aged ≥18 years and currently smoking in the United States (March 2019-April 2020). Participants uploaded photos daily that depicted smoking risk and protective factors they experienced over 21 days on either private Facebook or Instagram groups. Next, we conducted separate focus group discussions to explore the experiences of these factors among a subset of participants from each group. We analyzed participants’ photos, captions, and focus group transcripts and generated themes associated with smoking risk and protective factors.

**Results:**

We identified 6 major themes of risk and protective factors of smoking among TGE individuals: experience of stress, gender affirmation, health consciousness, social influences, routine behaviors, and environmental cues. We describe and illustrate each theme using exemplar photos and quotes.

**Conclusions:**

The findings of this study will inform future community-engaged research to develop culturally tailored interventions to reduce smoking prevalence among TGE individuals.

## Introduction

### Background

Cigarette smoking is the leading cause of preventable cancer, cardiovascular, respiratory, and other smoking-related illnesses and deaths in the United States [1]. Despite the decline in overall smoking rates in the US population over the past 50 years, there are persistent disparities in the prevalence of smoking among vulnerable populations [2]. Transgender and gender-expansive (TGE) adults—individuals who have a gender identity, behavior, or self-expression that is different from their sex assigned at birth—are twice as likely to smoke cigarettes than cisgender individuals [3]. Approximately 36% of TGE adults smoke cigarettes compared with 21% of heterosexual cisgender adults [3]. An estimated 1 million TGE adults live in the United States [4], which means that approximately 350,000 TGE individuals are at increased risk of developing smoking-related cancers.

The factors associated with increased smoking prevalence among TGE persons are complex and include TGE-specific minority stressors, including everyday discrimination (eg, experiencing denial of equal service, harassment, and physical assault due to TGE status) [5,6], structural discrimination (eg, housing, education, and work discrimination due to TGE status) [6], social determinants (eg, lower socioeconomic position and lack of health insurance in part due to employment discrimination), and alcohol and other substance use [6]. In addition, the tobacco industry targets its product marketing and advertising among lesbian, gay, bisexual, transgender, and queer (LGBTQ+) communities [7,8]. Compounding these risk factors, TGE individuals lack equitable access to health care and, therefore, face barriers in receiving smoking cessation interventions [9]. Research suggests that given the appropriate resources and opportunities, TGE adult smokers are just as likely as cisgender smokers to want to quit [9-11]. Despite experiencing multiple risk factors of smoking, receiving gender-affirming care earlier on recognition of TGE status and social support systems may be protective against smoking among TGE persons [12]. There is a critical gap in research on effective and culturally sensitive approaches to reduce smoking prevalence among TGE adults. Of 384 National Institutes of Health–funded sexual and gender minority research studies in 2018, 20% focused on TGE health and only 1% focused on tobacco use and health [13]. Furthermore, although there is increasing evidence of the effectiveness of using social media for HIV interventions among LGBTQ+ populations [14], there is limited research on the use of web-based social media platforms for tobacco-related research and smoking cessation interventions among TGE populations [15-17].

### Objectives

**Figure 1:**
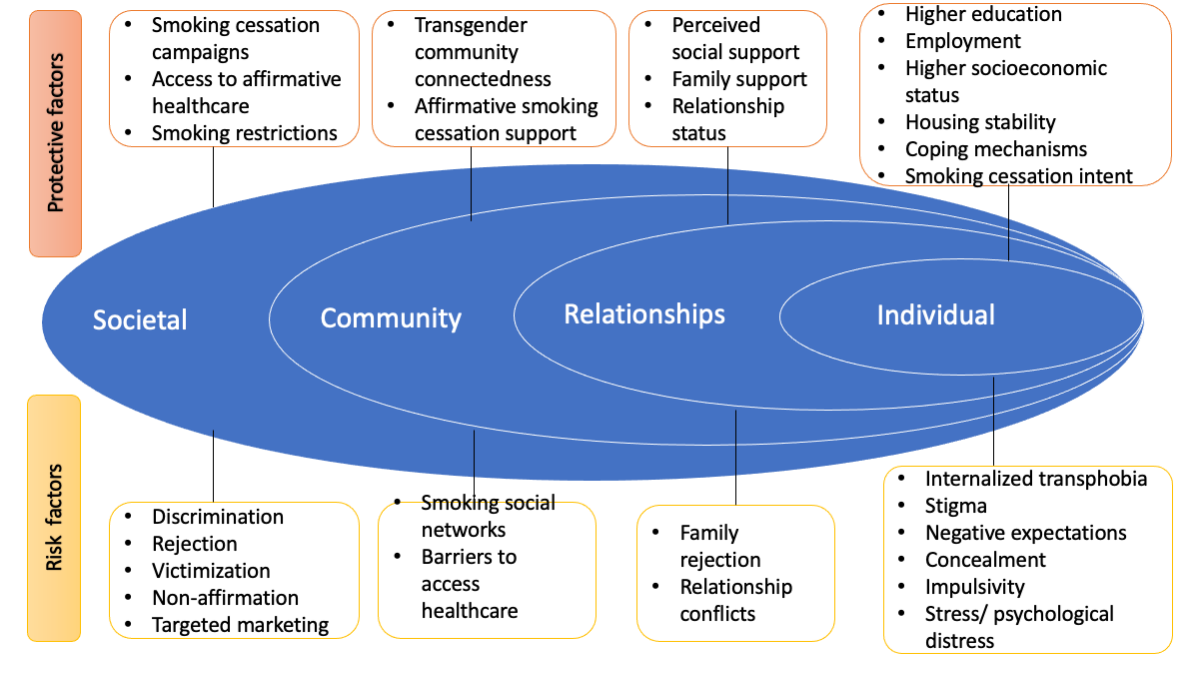
Conceptual framework.

The objective of Project SPRING is to examine TGE individuals’ experiences of smoking risk and protective factors in close-to-real time by using a photovoice approach whereby participants “identify, represent, and enhance their community through a specific photographic technique” [18,19]. Photovoice has been used in prior research to examine the social experiences of TGE individuals [20]. The rationale for using photovoice instead of existing approaches such as qualitative interviews or quantitative surveys are threefold. First, we aimed to obtain participants’ documentation of their experiences of risks and protective factors in close to real time to minimize recall bias associated with interviews and surveys. Second, photovoice enables the exploration of new or emergent risk or protective factors that are not limited by close-ended survey questions. Third, the photovoice approach involves multiple interactions with participants over time and enables the collection of multiple types of data from the same participants. Focus groups at one time point and cross-sectional surveys are limited in this regard. Our approach was guided by a community-based participatory research approach [21], where TGE persons were included as cocreators of knowledge in several aspects of the study, including data collection, analysis, and interpretation, to inform future research and interventions. This study approach empowers TGE persons to work collaboratively with the study team to understand factors influencing their smoking habits and generate evidence to inform future TGE-tailored smoking cessation interventions. This research is informed by an integrated framework of key concepts from the minority stress model, resilience framework, and socioecological model [22,23]. We conceptualized smoking behaviors among TGE persons to be influenced by both risk and protective factors across individuals, relationships, community, and societal levels (Figure 1). Findings from the real-world exemplars, phrases, meanings of smoking-related triggers, and protective factors from this research will serve as the foundation for designing culturally sensitive narrative messages to promote smoking cessation through social media among the TGE community.

## Methods

### Research Design

We used a qualitative research design using digital photovoice data collection, focus group discussions, and member checking among participants. The study was approved by the institutional review board of Harvard TH Chan School.

### Participant Eligibility and Enrollment

We enrolled a purposeful sample of participants who identify as TGE, live in the United States, have smoked at least 100 cigarettes in their lifetime, currently smoke cigarettes on one or more days in the past 30 days, use either Facebook or Instagram at least once daily, and were able to participate in English. Individuals who were currently quitting smoking, only used e-cigarettes or cannabis, did not smoke cigarettes, and were not able to participate using the English language were ineligible. We used Facebook and Instagram because of the high prevalence of use of these 2 platforms and increased exposure to tobacco-related messages on social media among LGBTQ+ populations [24,25]. The study enrollment was conducted between March 2019 and April 2020. Our initial recruitment was in a northeast metropolitan city, and we expanded the recruitment nationally in October 2019. We recruited most participants through paid Facebook and Instagram advertisements based on the best practices of engaging TGE populations on the web for research studies [26,27]. We also used Craigslist advertisements, snowball sampling, posting flyers at LGBTQ+ events and venues, and outreach through LGBTQ+ community organizations. Two study research assistants screened interested participants over the phone and consented participants who were eligible and agreed to participate in the study. Approximately half of the participants joined separate Facebook groups and half joined separate Instagram groups. The recruitment proceeded on a rolling basis, and participants within each group completed the photovoice collection within the same period of 21 days in their respective groups. There were between 2 and 9 participants per group (average of 5 per group). All participants in each group were given the same start date. Participants received gift cards as incentives for completing each phase of the study procedures (US $10-75 per phase; up to US $300 for completing all phases). We received messages from over 330 interested individuals and were able to contact 110 for screening over the phone or through Facebook messaging. Of the 110 individuals, 47 were eligible and enrolled in the first phase of the study. Of the 47 individuals, 44 completed photovoice data collection. Of the 44 individuals, 29 participated in the focus group discussion. Among these, 9 individuals participated in the member checking discussion.

### Study Procedures

#### Digital Photovoice Data Collection

We used the photovoice technique [18] to obtain participants’ close-to-real time personal experiences of risk or protective factors of smoking. Participants were asked to upload pictures and a brief caption of either risk or protective factors in relation to their smoking they experienced to a private study-specific Facebook group or on an Instagram group chat daily for 21 consecutive days. Participants’ information on the Facebook or Instagram groups could only be viewed by the participants within their group and the study team. Participants were encouraged to post at least 1 picture per day for at least 5 days per week to ensure a minimum level of participation in the study. They could provide comments on each other’s posts. To ensure their privacy and confidentiality, 2 study research assistants conducted training with each study participant to describe the study protocol and examples of the types of appropriate pictures, phrases, and comments that can be shared within their respective Facebook groups or Instagram group chats. Each participant was assigned a unique study ID code number not connected to any personally identifiable information.

#### Focus Group Discussion

After each group completed the photovoice data collection, we invited all participants to a focus group discussion with their respective group members. A PhD-prepared nurse researcher with extensive experience and training in qualitative research led the focus groups to research assistants. We conducted in-person focus groups among participants from a northeast metropolitan city and conducted web-based focus groups using the Zoom web conferencing platform (Zoom Video Communications) among participants who were recruited nationally. The number of participants across the focus groups ranged between 2 and 7. The duration of the focus groups ranged from 70 to 170 minutes. During each focus group, participants were first shown photos that they contributed within their Facebook closed private group or Instagram group chat. They were asked to work as a group to complete an image sorting task where they first categorized the images as either risk (things that make them want to smoke) or protective factors for smoking (things that help them resist smoking). Following sorting into risk and protective factors, we asked participants to describe each photo categorized as a risk factor in their own words. This was then repeated for each photo, which was categorized as a protective factor. The moderator provided guidance to the group and probed for the rationale for certain categorization decisions using the SHOWED questions as needed (What do you **S**ee here? What is really Happening? How does this relate to Our lives? Why does this condition Exist? What can we Do about it?). Participants’ final categories and names of each category as decided by the group’s consensus were recorded. The in-person focus groups were audio recorded, and for the web-based focus groups, we obtained an audiovisual recording and immediately deleted the video component. Following transcription by a professional transcriber, the recordings were deleted per institutional review board requirement for participant confidentiality and privacy.

#### Member Checking

Following the study team’s preliminary analysis of the photos, captions, and focus group transcripts, we invited participants from all the Facebook and Instagram groups to attend a web-based member checking discussion to obtain insights on their interpretation of the risk and protective factors of smoking behaviors, identify the most important factors to address in future interventions, and discuss potential approaches for culturally tailored interventions to reduce smoking among TGE persons. A total of 9 participants were able to attend the member checking focus group. The member checking participants were not all from the same initial Facebook or Instagram group but were members of 5 different groups. This member checking discussion was conducted over 67 minutes over Zoom web conferencing, and we obtained an audio recording. Following transcription by a professional transcriber, the audio recording was deleted.

### Analysis

Photos, captions, and focus group transcripts were uploaded to NVivo 12 (QSR International) by the study team for coding. Analyses of photos and captions were conducted in conjunction with the participants during the focus groups (photo sorting and labeling of risk and protective factors using participants’ own words). After completing the focus groups, the study team synthesized the data into related categories (eg, weather, climate, and physical environment were combined into the same category). Furthermore, 2 research assistants coded the transcripts from the 2 focus groups to ensure agreement in an initial coding guide. Each research assistant then coded the remaining focus group transcripts individually. Emerging codes or questions were resolved by discussion with the study team, and the codebook was updated following the discussion. The final codebook is available as Multimedia Appendix 1. We categorized factors as being risk or protective factors; occurring at individual, relationship, or community levels; and specific factors (eg, taste of cigarettes, minority stress, and coping mechanisms). We then organized the individual codes into major themes of risk and protective factors. This study followed the Consolidated Criteria for Reporting Qualitative Health Research. On the basis of recommendations from the literature [28], we estimated that 3-6 focus groups would be sufficient for theme saturation.

## Results

### Participant Characteristics

Saturation of themes was achieved after conducting 7 focus groups between March 2019 and April 2020 with a total of 47 participants. The mean age of the participants was 26 (SD 8.4) years. Approximately half of the participants (25/47, 53%) were identified as nonbinary or gender nonconforming, 15% (7/47) were identified as male, trans male, or trans men, 17% (8/47) as female, trans female, or trans women, and 15% (7/47) identified with other gender identities. Most of the participants identified as nonheterosexual; half of the participants (22/47, 47%) identified with multiple sexual orientations, 17% (8/47) as bisexual, 13% (6/47) as queer, 11% (5/47) as pansexual, and 9% (4/47) as gay or lesbian. Most (40/47, 85%) of the participants were non-Hispanic, with 55% (26/47) identified as White, 13% (6/47) Black, and 32% (15/47) other racial or ethnic identities. Approximately half of the participants (26/47, 55%) were daily smokers, and half were occasional smokers in the past 30 days. Additional details of participant characteristics are summarized in Table 1. We compared the characteristics (eg, age, sex assigned at birth, gender identity, and sexual orientation) between participants in the northeast city and those recruited nationally, and there was no significant difference.

**Table 1:** Participant characteristics (n=47).

Characteristics		Values
Age (years), mean (SD)		26.0 (8.4)
**Sex assigned at birth,** **n (%)**		
	Male	17 (36.2)
**Gender identity,** **n (%)**		
	Male, trans male, or trans man	7 (14.9)
	Female, trans female, or trans woman	8 (17)
	Nonbinary or gender nonconforming	25 (53.2)
	Other	7 (14.9)
**Sexual orientation,** **n (%)**		
	Heterosexual	2 (4.3)
	Gay or lesbian	4 (8.5)
	Bisexual	8 (17)
	Pansexual	5 (10.6)
	Queer	6 (12.8)
	Multiple identities	22 (46.8)
**Race,** **n (%)**		
	White only	26 (55.3)
	Black only	6 (12.8)
	Other	15 (31.9)
**Ethnicity,** **n (%)**		
	Hispanic	7 (14.9)
**Smoking status,** **n (%)**		
	Occasionally smoke	21 (44.7)
	Smoke daily	26 (55.3)
**Other tobacco product use in past 30 days,** **n (%)**		
	Electronic cigarettes	16 (34)
	Cigars	10 (21.3)
	Pipe	4 (8.5)
	Hookah	8 (17)
	Snus	1 (2.1)
	Kreteks	6 (12.8)
**Education,** **n (%)**		
	High school or below	12 (25.5)
	Some college or associates degree	25 (53.2)
	Bachelor’s degree or higher	10 (21.5)
**Employment,** **n (%)**		
	Employed	25 (53.2)
	Unemployed	7 (14.9)
	Homemaker	1 (2.1)
	Student	8 (17)
	Disabled	4 (8.5)
	Other	2 (4.3)
**Income (US** **$** **),** **n (%)**		
	<10,000	17 (36.2)
	10,000-19,999	14 (29.8)
	20,000-39,999	4 (8.5)
	40,000-59,999	6 (12.8)
	60,000-79,999	2 (4.3)
	80,000-99,999	0 (0)
	≥100,000	4 (8.5)
**Health insurance, n (%)**		
	Plan purchased through employer	12 (25.5)
	Plan purchased on their own	10 (21.3)
	Medicare	4 (8.5)
	Medicaid or another state program	12 (25.5)
	Tricare	1 (2.1)
	Some other source	2 (4.3)
	None	6 (12.8)

### Risk and Protective Factors

#### Overview

**Figure 2:**
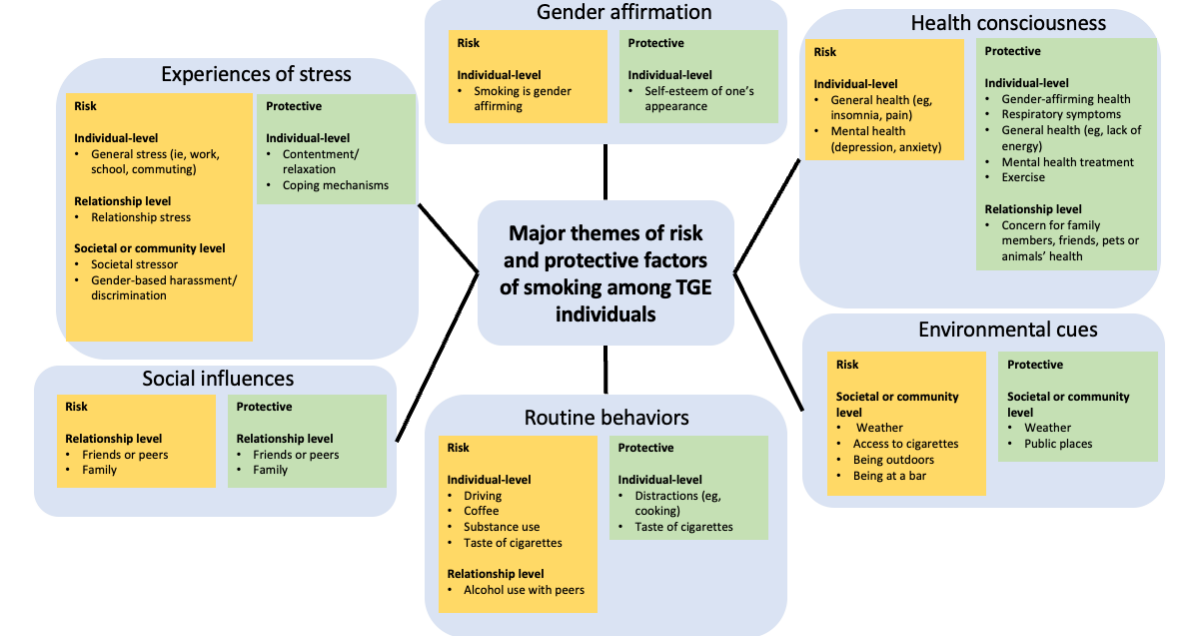
Smoking risk and protective factors among transgender and gender-expansive individuals.

We identified 6 major themes of smoking risk and protective factors: (1) experiences of stress, (2) gender affirmation, (3) health consciousness, (4) social influences, (5) routine behaviors, and (6) environmental cues. Risk factors were described more frequently than the protective factors. In addition, participants experienced certain factors as risk factors in some circumstances and as protective factors in other circumstances (eg, friends or peers [social influence] were described as risks by some participants, whereas others described having friends or peers who encouraged them to quit smoking). Stress was a cross-cutting factor that occurs at the individual, relationship, and community levels. We describe and illustrate each theme, specific codes within each theme, organized into risk versus protective factors, and indicated which levels the factors were coded in Figure 2. We used they/them/their pronouns in the following section, although individual participants may use other preferred pronouns to present the results. We reviewed the data to compare the risk and protective factors from participants in the northeast city versus national participants, and there was no discernible pattern to distinguish between the 2 sets of participants, with the exception that 2 groups from the national sample had discussed experiences related to the COVID-19 pandemic that were not present in the northeast city participants (because they were recruited before the pandemic).

#### Experiences of Stress

Participants described a variety of stressors linked to smoking behavior. Some participants highlighted their experiences of minority stress related specifically to their gender identity (eg, stigma associated with their gender identity and concealment) that were associated with their smoking. For instance, one participant recounted a fellow colleague who identified as TGE being harassed by a customer:

One of my fellow trans coworkers was harassed, screamed at, and intentionally misgendered as well as received a torrent of slurs from a woman that came in today and that definitely made us all want to smoke.

Another participant shared a photo of being in an office with their colleagues and having to conceal their identity from them and the following caption:

I work in an office with all men and most of them are trans/homophobic but almost none of them know that I’m gay or trans and f* do they test my patience every day and that’s what makes me want to smoke.

Work, school, commuting, transportation, financial situation, housing, and relationship conflicts were factors that triggered increased smoking for some participants. For instance, one participant shared a stock photo of 2 people quarreling to reflect on a conflict with their partner and this accompanying caption:

We don’t fight much, but I’m a very confrontational person so not getting to finish the argument right away stressed me out to the point of wanting to smoke.

Participants described societal stressors such as homelessness, the economy, and lack of access to affordable mental health care as risk factors for smoking. For example, one participant posted a drawing depicting homelessness as a source of their stress and smoking with the following caption:

This pic represents all the economic issues, personal and larger, that stresses me out. I know there are lots of healthier ways to deal with my financial anxieties...BUT Cigarettes are cheaper than therapy.

Regarding coping behaviors to reduce feelings of stress, participants described using various strategies including achieving relaxation or contentment, using humor, or exercising. For example, one participant shared about using humor to help avoid smoking:

Anything that makes me laugh that’s like kind of making fun of anything is like, helpful and not smoking actually, so it feels protective to me because humor helps me get through all the horrors of the world that we’re living in.

#### Gender Affirmation

Participants described smoking as one way to affirm their gender identity and experience gender euphoria. For instance, one participant shared a photo where they were smoking in their car while driving and a caption:

I’m a very good driver I believe, which is a stereotypically “masculine” thing, and smoking is seen by some people as masculine, as well. It’s affirming in a weird way.

Conversely, feelings of self-esteem of one’s appearance were associated with not wanting to smoke. One participant shared an occasion of dressing up and not wanting to ruin their appearance because of smoking:

I sometimes dress up, put on the whole makeup, wear some nice type of deal. And it kind of ruins that effect when you smell predominantly like smoke and not nice new perfume...

#### Health Consciousness

Participants viewed receiving gender affirmation–related health care and health care provider advice to stop smoking as a motivating factor to not smoke. For instance, one participant shared a picture of having completed top surgery and a caption stating that they wanted to quit smoking so that the scars would heal better. Another participant described being receptive to their doctor’s advice:

If my doctor told me that I had to stop taking estrogen, this happened and this happened and I didn’t stop smoking, you know I’d stop smoking.

General health was a factor that participants experienced as risks for smoking in some situations and as protective in other situations. General physical health issues, such as insomnia and pain, or mental health issues, such as anxiety and mood, were identified as potential risk factors for smoking. Having respiratory symptoms, seeking mental health therapy, and practicing self-care were also described as protective against smoking. One participant shared a photo of a cup of tea and stated in the caption that having a sore throat made them want to drink tea and avoid smoking. Another participant described insomnia and anxiety as triggers of smoking:

I feel like sleep and/or lack of sleep and anxiety are pretty close together. I feel like people tend to feel more anxious when they’ve maybe not slept very well. And cigarettes are one way of like coping with that and sort of addressing that.

Participants were concerned about the health of family members, pets, or animals around them if they smoked, which motivated some participants to avoid smoking. One participant posted a photo of being with young children and wrote:

Being with my kids makes me not want to smoke. Their health and my own makes me always reconsider my choices.

Another participant shared how they would not smoke near their pet to avoid harming their pet because of secondhand smoke:

I remember reading a bunch of articles about how secondhand smoke affects pets. And I really love my cat and I don’t want him to get any ill effects from me smoking. So, whenever I do, I try to do it on the porch and I try to lock him out, which he hates...

#### Social Influences

Social influences were described as risks in some circumstances and protective factors in others. Being in the company of friends and peers in social situations where smoking and other substance use were present were risk factors for smoking. One participant explained the following in the focus group:

...there are things that make me want to smoke, and seeing anyone else with a cigarette or hearing anybody else talk about having a cigarette is one of those things.

Positive family pressure and friends or peer norms to not smoke were associated with avoiding smoking. One participant posted a photo outdoors with their partner and described their partner’s disapproval of their smoking in the caption:

My best friend/life partner HATES when I smoke, so thinking of her makes me want to quit.

#### Routine Behaviors

Routine behaviors such as driving or drinking coffee were viewed as risk factors for smoking. For instance, one participant shared a photo of sitting in their car and a caption of being triggered to smoke whenever they drive.

The taste of cigarettes was another factor within the theme of habit that increased smoking (risks) for some participants but deterred others from smoking (protective). One participant shared that the taste of finishing one cigarette prompted lighting up another cigarette. Another commented on cigarettes being *nasty* and that made them not want to smoke.

Substance use, including alcohol and cannabis, was also discussed as a risk factor for smoking cigarettes. For example, one participant shared a picture of a can of beer while playing a drinking game that led to their desire to smoke a cigarette:

Playing a drinking game tonight. Alcohol always makes me want a cig. Especially since one of my roommates smokes too and I know he’ll go out for one at some point.

Conversely, having distractions was helpful in keeping participants’ hands and minds occupied and avoiding smoking. These distractions included cooking a healthy meal, working on a school assignment, occupying their hands by playing a musical instrument, viewing positive images, and watching entertainment shows.

#### Environmental Cues

Participants described environmental cues that prompted their smoking, including being outdoors, experiencing either good or bad weather, easy access to cigarettes, images related to smoking, or being in a bar. One participant shared a photo of a rainy day outside their home and a caption:

Cold, wet, boring, and grey day. Nice time for a cigarette.

Conversely, some participants described environments or situations that discouraged them from smoking, including places where they were not able to smoke, such as at a transit stop and bad weather, which prevented them from going outdoors to smoke. One participant posted a picture of being at a picnic in a public park and captioned it as follows:

I love to have a smoke while walking around. but it was a public park, so I waited until I got home.

## Discussion

### Principal Findings

This study described a comprehensive set of risk and protective factors occurring at multiple levels among a sample of TGE smokers based on their real-world experiences and through an analysis of a combination of photos, captions, and focus group discussions. Participants in this study reported more risk factors for smoking than the protective factors. This was understandable because they were current smokers and did not attempt to quit smoking at the time of the study. This information is unique and essential for understanding the circumstances and reasons for smoking and for avoiding smoking among TGE individuals. Most themes of risk and protective factors described by the participants were consistent with prior literature on the determinants of smoking among LGBTQ+ populations [5,6]. However, to our knowledge, this is one of the first few studies to identify unique factors associated with smoking among TGE individuals, including gender affirmation and gender affirmation–related health care.

These study findings help us understand the important factors that underlie smoking habits among TGE individuals, which can serve as the foundation for future research and smoking cessation intervention design. First, there is a need for future research on the underlying factors linked to smoking or not smoking that either occur more frequently or are unique among TGE populations. Although prior literature reported correlations between receipt of treatment for gender affirmation and smoking behavior or smoking cessation [29,30], this study adds to earlier research by documenting participants explaining in their own words how gender affirmation and gender euphoria were related to their smoking behaviors. This study identifies factors that focus on preventing smoking initiation and promoting cessation among TGE populations that have not been previously described, such as gender affirmation and receiving gender affirmation–related health care. Future work to pretest the relevance and acceptability of health messages that also address the theme of gender affirmation to promote smoking cessation among TGE populations will be needed. Second, the images, phrases, and stories from TGE participants’ lived experiences may inform the design of antismoking messages drawn from authentic testimonials that are salient and culturally responsive to TGE individuals. For instance, participants described real-life situations of avoiding smoking by practicing self-care in various ways, which could be adapted as tips for TGE individuals in future antismoking messages. Third, participants described factors that were both risk and protective factors depending on the circumstances and revealed that certain factors occurred across individuals, relationships, and societal or community levels. The findings support the use of a multilevel framework and systems change approach to examine and address smoking holistically among TGE populations. Fourth, some participants affirmed the value of the collaborative research approach and provided feedback and enthusiasm for contributing to the research and future intervention design as community advisors. We will use a community-based participatory research approach by meaningfully engaging with TGE individuals as coequal partners and advisors during the next phase of this research and in future intervention design.

These findings are corroborated by several factors in the conceptual framework (Figure 1). However, some of the factors that we anticipated would be important drivers of smoking were absent in the data obtained from this research. These include the tobacco industry’s marketing [7,8] and smoking cessation campaigns, which we hypothesized as potential influences but were not mentioned by participants as risk or protective factors of their smoking behaviors, respectively. A few themes that emerged in the study were not accounted for in the framework. These included health consciousness for others, such as family members and pets, routine behaviors, and environmental cues.

A few themes reported among TGE individuals resemble, at the surface, factors that have been previously described in the general population, including stress, social influences, routines, and environmental cues. Although these factors are not unique to TGE individuals, the *contexts* and *frequency* of experiencing these factors may differ meaningfully between the TGE and non-TGE populations. For instance, the underlying structural discrimination of TGE populations may mean that they experience greater economic stress and financial difficulties [6] than non-TGE populations, which, in turn, widens the disparity in smoking behavior among TGE populations. The issue of the social determinants impacting TGE populations and their linkage with their experience of stress was highlighted by some participants in our study. Future research exploring the similarities or differences in risk and protective factors between TGE and non-TGE populations will help to address questions about the contexts of experiencing these factors.

There were several lessons learned from the use of the digital photovoice approach within closed social media groups. A few participants reported during the member checking discussion that being in the study provided peer support and they appreciated being in a group that understood their experiences as TGE persons. Some participants described how the action of uploading a photo daily related to their smoking behavior helped them to keep track of their smoking use, and they became more aware of the motivations to smoke. Participants reported that the groups provided a safe space that allowed them to connect and communicate with other participants who had similar experiences. For instance, one participant commented that “with these people I can do anything, including try to quit smoking.” The study procedures were generally viewed as acceptable and not overly burdensome. We note that this study was not designed as a smoking cessation intervention, and participants did not intend to quit smoking at the outset of the study. However, feedback from the participants suggested that the use of social media peer groups may be a promising component of group-based support and interventions to reduce smoking among TGE populations. Compared with previous research that used different approaches (eg, close-ended cross-sectional surveys [3,5,6,11,30], focus groups among participants in a single geographic location [9], and electronic health record data [12,29]), the photovoice approach in this study has the advantage of obtaining rich and contextual visual data of experiences of smoking risks and protective factors in close to real time (vs recall from the past), detailed exploration of a broad range of factors impacting smoking behaviors using the participants’ own words (vs close-ended survey instruments), and the ability to combine multiple sources of data collected from the same participants over time and geographic areas (vs one-time focus groups from one geographic location).

### Limitations

This study has a few limitations. Owing to the limited sample size of participants from racial and ethnic minority backgrounds, we were unable to fully explore whether experiences of multiple axes of discrimination by gender identity, race, and ethnicity intersect in experiences of risk and protective factors among TGE individuals from multiple minority backgrounds. Efforts to increase enrollment of TGE individuals from multiple minority backgrounds in future work will yield data to critically address research questions related to intersectionality among these individuals. Although this study yielded a comprehensive set of risk and protective factors, the study data were not representative of the experiences of the broader TGE population who smoke. However, this work will inform the design of future survey research among a larger national sample of TGE participants to assess the frequency of encountering these smoking risk and protective factors and appropriate strategies to address these factors in reducing smoking among TGE individuals. This study relied on participants who were motivated and comfortable with documenting and sharing their experiences of smoking risk and protective situations in the form of photos to their group. Although we did not receive feedback from participants that this deterred their sharing of certain experiences, we acknowledge that this limitation may mean that certain sensitive topics related to smoking specifically may have been omitted. Future research may include an approach in which participants can submit photos individually instead of within a group.

### Conclusions

To summarize, this study identified real-world risk and protective factors among TGE individuals who smoke and collect rich visual representations and participants’ own words in labeling risk and protective factors. The long-term goal of this research is to reduce disparities in tobacco use and related health disparities among the TGE populations. The lessons learned from this study approach and rich data will inform future community-engaged research for designing a culturally responsive intervention to address these factors with TGE community members as coequal partners.

## Appendix

Multimedia Appendix 1 Smoking risk and protective factors.

## Abbreviations

LGBTQ+: lesbian, gay, bisexual, transgender, and queer
TGE: transgender and gender-expansive
