# Supplementary material for: Smoking Protective and Risk Factors Among Transgender and Gender-Expansive Individuals (Project SPRING): Qualitative Study Using Digital Photovoice
Source: JMIR Public Health Surveill. 2021 Oct 6;7(10):e27417. doi: 10.2196/27417 (PMC8529476; doi:10.2196/27417)
Supplement: Multimedia Appendix 1 [file publichealth_v7i10e27417_app1.docx]

**Appendix A. Smoking Risk and Protective Factors**

|  | **Exemplar Picture/Caption** | **Exemplar Quote** |
| --- | --- | --- |
| *Experiences of stress:*  Minority Stress (Individual) (R) | *[Picture omitted because of identifying information]*  Been on the phone for 10 minutes with the insurance person for my top surgeon and she can't even find my chart.  *[Picture omitted because of identifying information]*  My face today. Was assaulted again last night, so all I want to do is smoke and disappear. It's difficult to trust anyone in my life, when they all keep doing things like this. My interned connections seem more solid, but part of me wonders if that's just because we haven't met in person yet. I know there are genuinely decent people in the world, but when so much of the world rejects everything I am, it's hard to keep hoping. | “This is like a weirdly specific one, because like I said, I was just looking for a house to rent and stuff, and I had to meet with a bunch of realtors and landlords and stuff. And that’s like normal life stress like most people at some point in their lives will go through. Something like that, right? But I definitely experienced specific stress that I feel like I probably wouldn’t have experienced if I wasn’t trans or like gender nonconforming. I just remember I’m very conscious - usually I dress however I want. I am very gender neutral presenting usually and I’ll wear some fun whacky clothes and I don’t really care to look like - to look specifically male or specifically female. Doesn’t really bother me either way. But when I went to meet with landlords and stuff, I pulled my hair back. I wore a button-up shirt. I dressed like a man, man.” |
| *Experiences of stress:*  Minority Stress (Relationships) (R) | *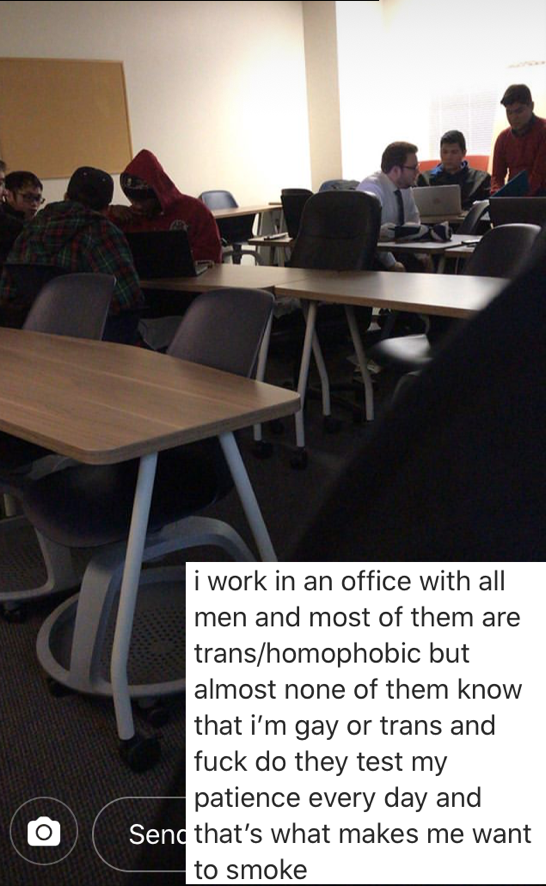* | “But, yeah. When it does happen, man – just because sometimes it’s like – that specific instance, one of – that person was publicly calling out one of my friends, and I was like, can you just not? And they were like, what you doing over here you fucking tranny? And I’m like, oh, gee, thanks, you – I’m just telling you not to be an asshole, you didn’t have to throw that in there. And then – yeah. Just probably the pain, I think, just makes you wanna smoke a lot” |
| *Experiences of stress:*  Minority Stress (Social Determinants) (R) | 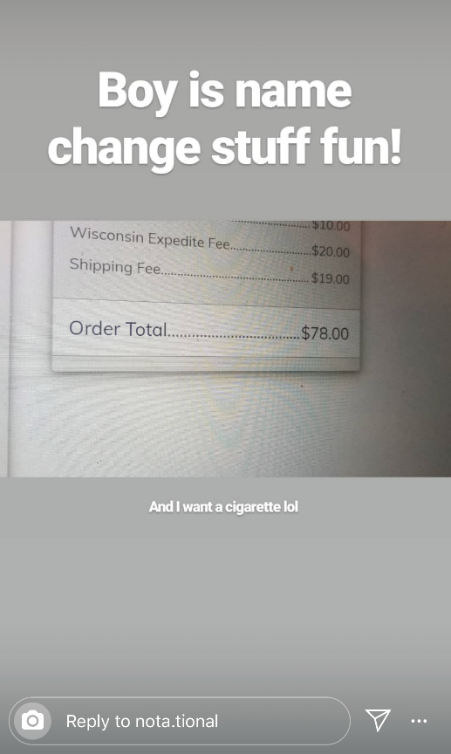  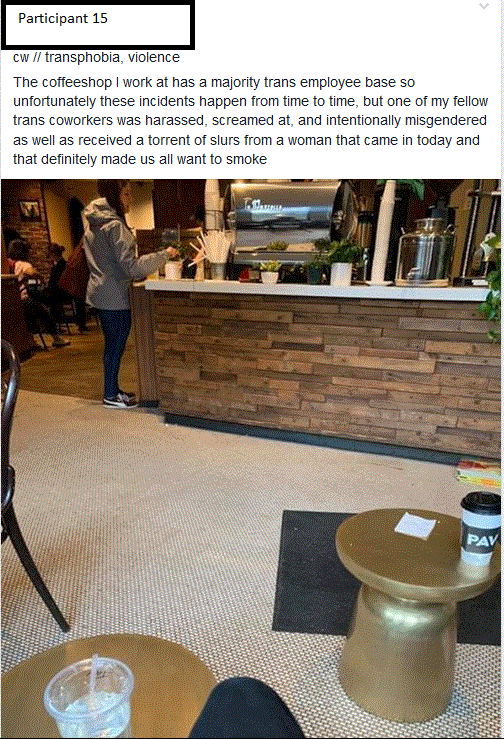 | “And besides, on the macro level – on the micro day-to-day sort of level at work, you can’t really just stop everyone who misgenders you. You have to just kind deal with it. If you have trouble with it, you can’t really make a big thing out of it.” |
| *Experiences of stress:*  Relationship Stress (Risk [R]) | 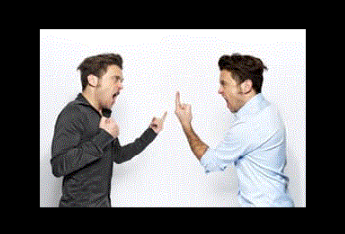Overly-dramatic stock image but today i got into a fight over text with my boyfriend (uses they pronouns). It wasn't over anything particularly serious but everytime we get into an argument and I make a point that makes them mad, they basically run away from the situation and it makes me unable to say anything. We don't fight much, but I'm a very confrontational person so not getting to finish the argument right away stressed me out to the point of wanting to smoke. I haven't done so today but that could change [Cold head emoji] | “Yeah. Yeah. Exactly. And social alienation in particular, because I feel like having time with your friends and your family to build space for solidarity and love is something that cigarettes attempt to - it’s like trying to fill that hold when we aren’t allowed the time to do what we really ought to be doing.” |
| *Experiences of stress:*  Stress (Individual) (R) | 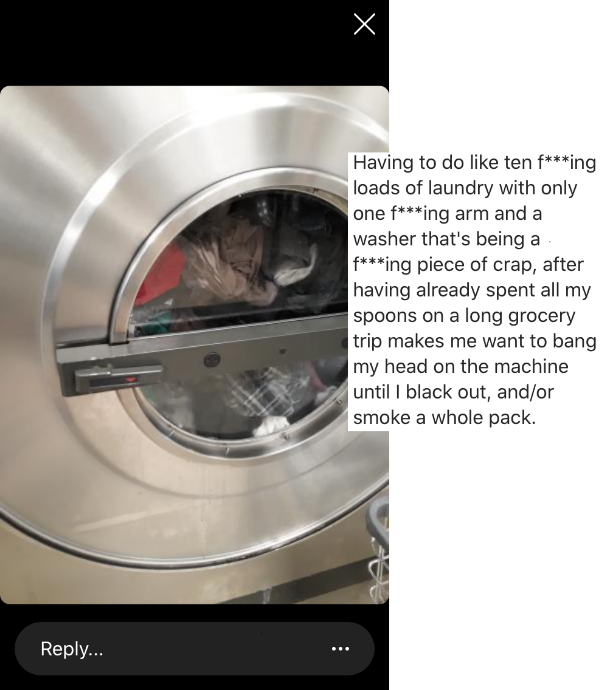 | I mean, they’re really just stressors. Most of these things are just subcategories that would fit into stressors. School, work. These things. Transportation, weather. Things that are less convenient for our lives make us want to smoke more. So that’s why these are all risk factors, because they’re basically just groups of things in our lives that are uncomfortable or inconvenient. (Individual) |
| *Experiences of stress:*  Stress (Social Determinants) (R) | 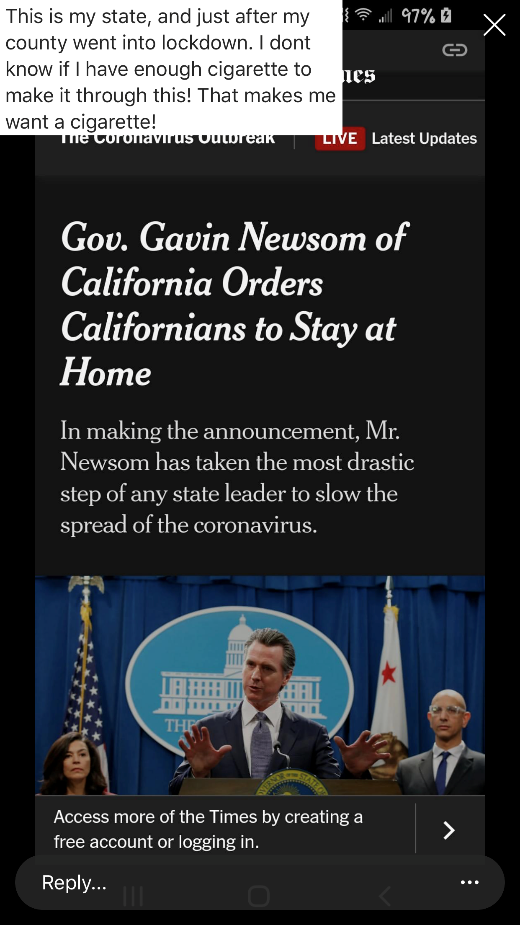  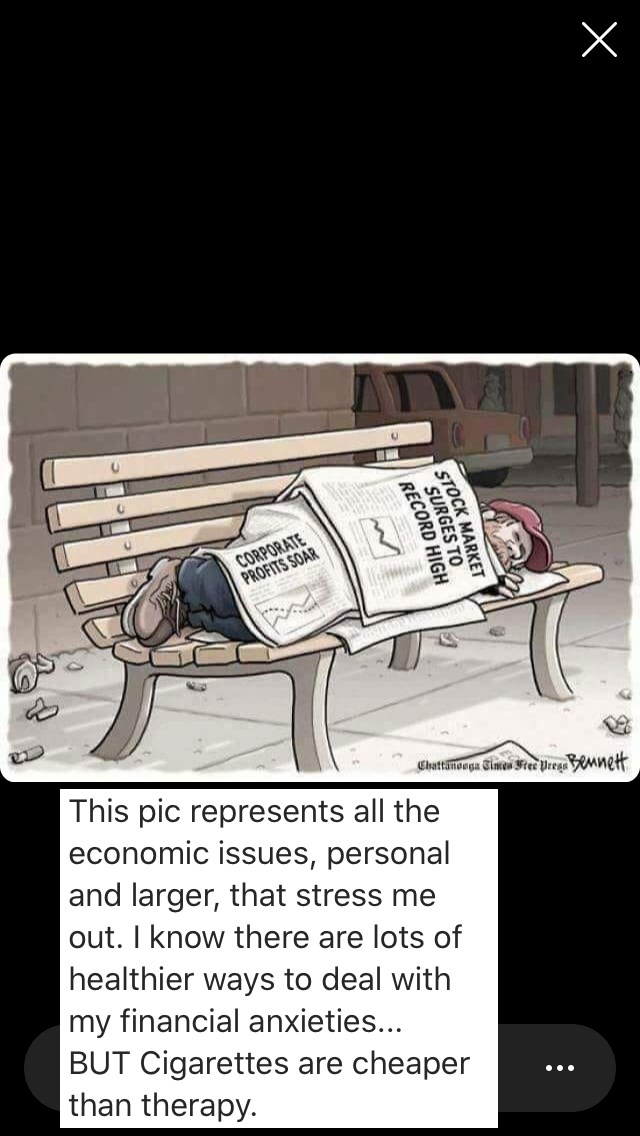 | “I was going to say just like overall, most things are related to like how our society functions, aka like capitalism, not to be all - but like exhausting yourself to work, school, finance. And smoking is a way to cope with it, which makes sense that coffee and alcohol are a thing that’s interrelated because I know for me, coffee will make the pick-me-up from the cigarette better. Alcohol - smoking will like drown.” |
| *Experiences of stress:*  Coping Mechanisms (Protective [P]) | 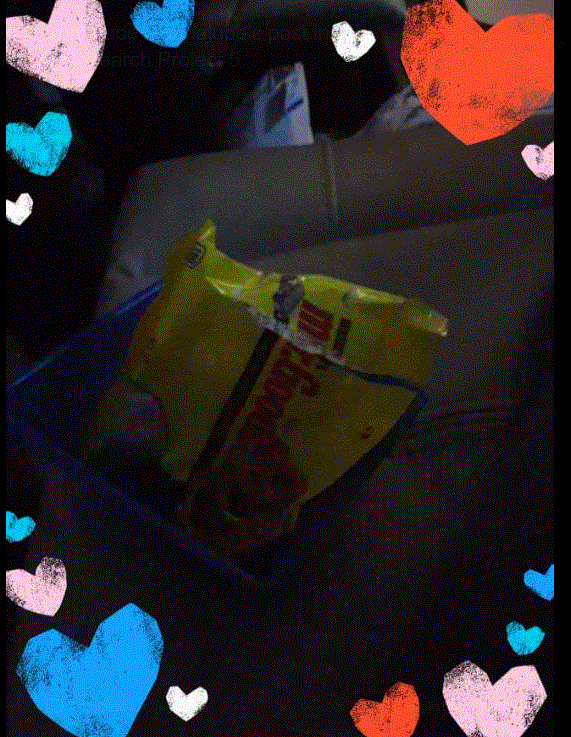  “I haven't been smoking recently... mostly because I can't width the obnoxious new laws passed and the COVID-19 quarantine going on rn...but I've been finding myself eating more and more especially whenever I crave cigarettes. I hate hate hate it and it makes me insanely depressed. I used smoking as a way to avoid binging or thinking/overthinking about food...but I guess this is how I avoid cigarettes..?” | “Anything that makes me laugh that’s like kind of making fun of anything is like, helpful and not smoking actually, so it feels protective to me because humor helps me get through all the horrors of the world that we’re living in.” (P) |
| *Experiences of stress:*  Contentment/Relaxation (P,R) | 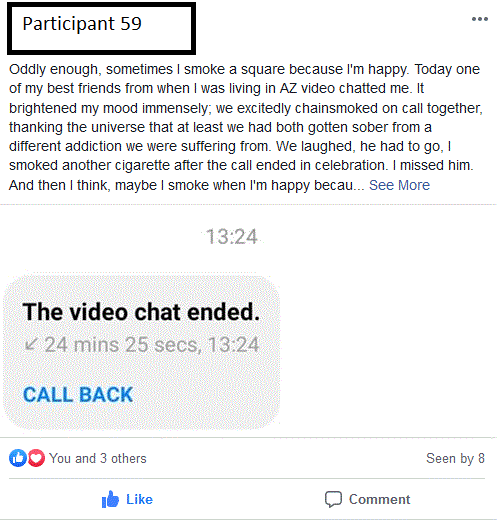  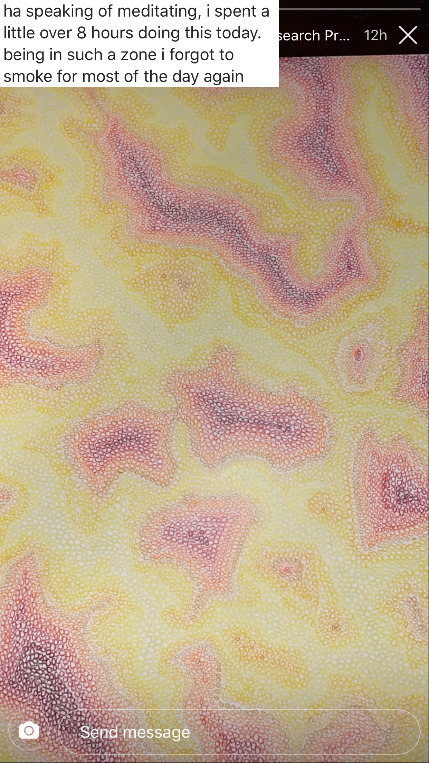 | “Yeah, just like when - sometimes you just want to smoke when it’s good times, like it’s there for the bad times. It’s there for the good times.” (R)  “There’s comfort in it though, no? I mean, for me, there’s like some comfort in it. And I have no control in the world and I know things are bad for me and it’s perpetual and I’m like yeah. I’m making choices right now. This choice is not good for me, and I’m just going to do it anyways. But it’s comforting, because like, I get to make that choice, and I do get some satisfaction just from that, if that makes sense.” (R)  “I think even though - because in the risk folder, we also had like a contentment section, so it definitely can be both. But I feel like this suggests - it’s more of a protective thing usually, and I feel like it definitely is for me. Like sometimes when I’m having a good time and just relaxing or whatever, I feel like I want to have a cigarette because it just seems like a nice time to. But that’s also when it’s easiest to resist, especially given that cigarettes cost money. I feel like sometimes I do that and then it’s like yeah, but if I smoke one now, something is going to piss me off later and I’m not going to have a cigarette then, and then I’ll be annoyed, so I wait. So I don’t know.” (P,R) |
| *Experiences of stress:*  Health Behaviors (P) | 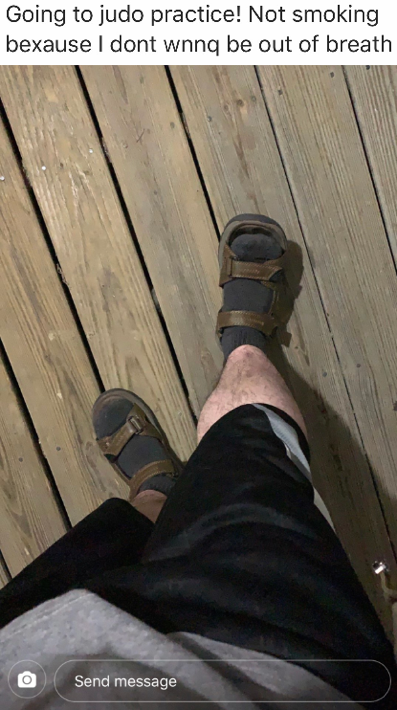 | “Yeah, exercising - smoking makes it really hard to exercise. You can’t really do both at the same time, or even close proximity to each other and not just have a bad time.” |
|  |  |  |
| *Gender affirmation:*  Gender affirming (R) | 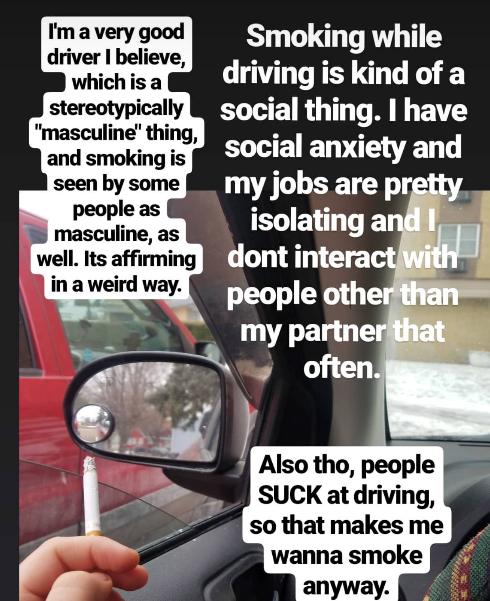 | “I mean, to me, I think it was pretty obvious that there were definitely gender or prejudice related to gender kind of elements that came into play and were risk factors for people, or someone I think - so one of them mentioned that like smoking makes them feel more masculine, which I definitely heard from other - like masculine-sector people say that it lowers their voice and they like it looks more masculine and this and that. I always feel tougher with a cigarette in my hand, which also falls into like personal safety, which is obviously a big issue for a lot of trans people, so yeah.” (R) |
| *Gender affirmation:*  Self-esteem (P) | 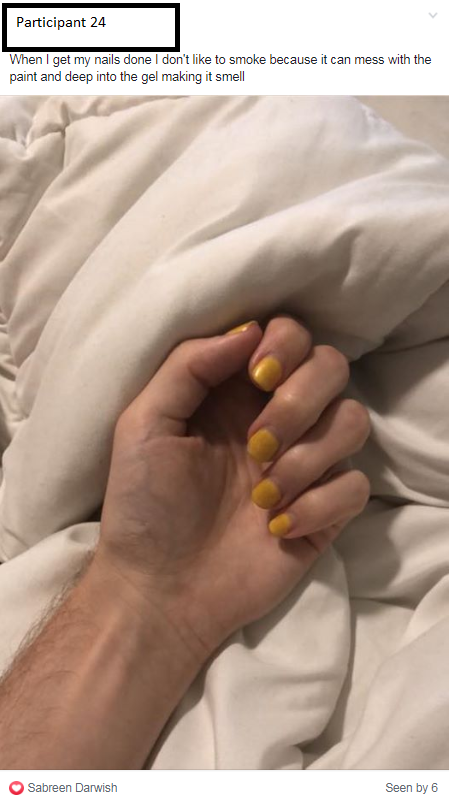 | “This isn’t mine but it’s kind of a mood. I sometimes dress up, put on the whole makeup, wear some nice type of deal. And it kind of ruins that effect when you smell predominantly like smoke and not nice new perfume, or whatever. So, I kinda feel this one. It was working as a makeup artist for a client and can’t smell like smoke, so I haven’t smoked today. So, I guess, self-esteem kind of.” |
|  |  |  |
| *Health consciousness:*  Respiratory Symptoms (P) | 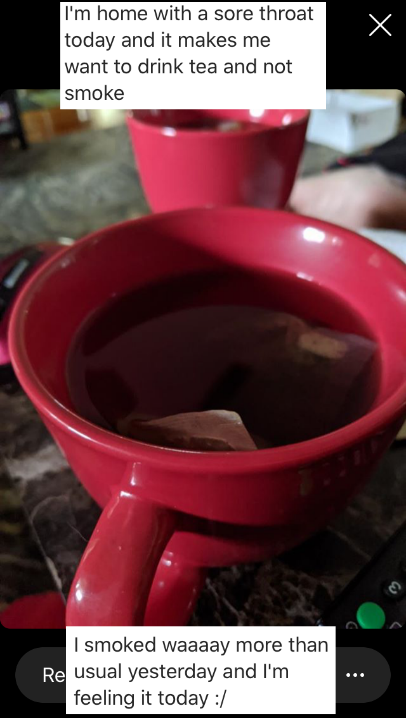 | “I kind of use my lungs for a lot of walking, so that was also like the past - I’ve been shooting a lot of historic battlefields, things like that. I’m working on like a documentary, so I’ve been out like miles of hiking, all these big fields. And they’re not just fields. It’s out in the woods and all. It’s a lot of mountainous terrain on some of that stuff. And honestly, that walking makes me personally smoke way less. Like way less.” |
| *Health consciousness:*  General Health (P,R) | 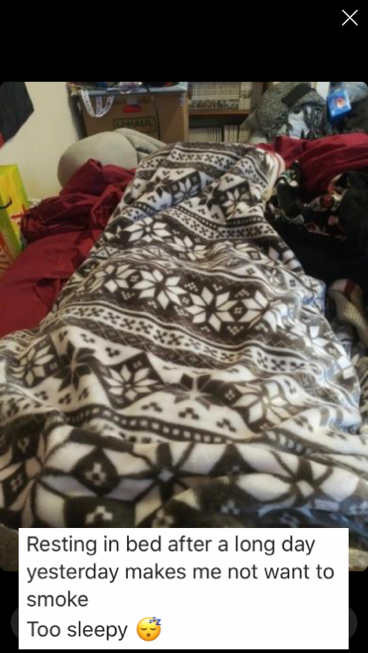 | “I feel like sleep and/or lack of sleep and anxiety are pretty close together. I feel like people tend to feel more anxious when they’ve maybe not slept very well. And cigarettes are one way of like coping with that and sort of addressing that.” (Risk) |
| *Health consciousness:*  Mental Health (P,R) | 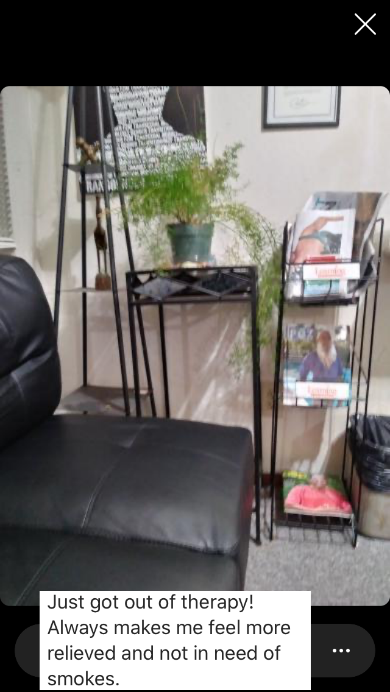 | “Like sometimes smoking helps me chill. I’m going to like, dissect what’s happening. But I could do that without a cigarette and just like sit outside for a second or something. And why I feel like that is helpful in my situation or relaxing or like there are a lot of other things in my life that are like immensely more relaxing than smoking a cigarette, but I still choose to have a cigarette over taking a bath or drinking some tea and like why that. And then it’s like there obviously is some sort of like bodily addiction, but like to what extent is it really just like my brain being all right. I mean, I know - and that is what it is, and that’s what’s hard.” |
| *Health consciousness:*  Gender affirmation-related Health (P) | 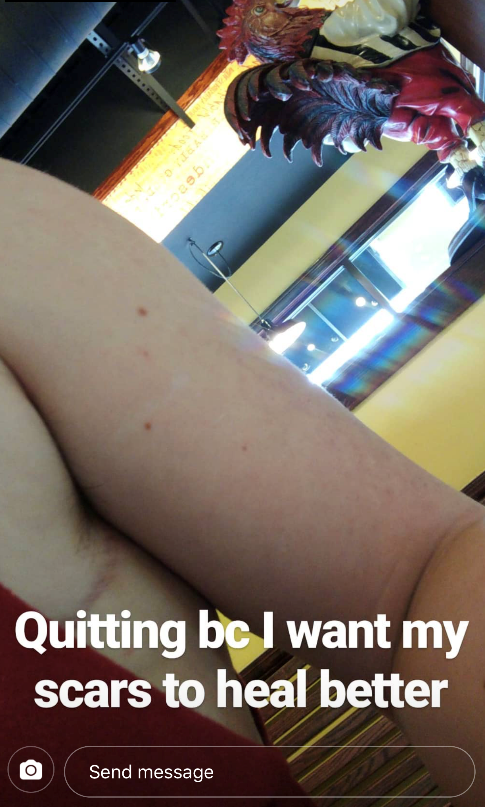 | “But transition-related health very specifically. Because I can sort of just like, okay, lung cancer, 50-50. But [laughter] that’s way more long term, theoretical, and stuff like that. I can tell myself that that’s maybe not gonna happen, or gonna happen way late in my life when everything’s shitty anyway. But when it’s something about transitioning, or whatever, then yeah, that’s important and very present now.”  *Alternate Options*  “Just to be healthier, I wouldn’t stop smoking. I don’t care. But if my doctor told me that I had to stop taking estrogen, [crosstalk] this happened and this happened and I didn’t stop smoking, you know I’d stop smoking.”  “Yeah. Whenever I’m thinking about something that’s important about health factors for transition, then I’m like – that’s a big protective factor.”  *In the context of anti-smoking PSAs “*My skin isn’t falling off yet and I don’t have emphysema, so I can’t relate to that being scary. But I can relate to not having estrogen.” |
| *Health consciousness:*  Concern for others health: (P) | *[Picture omitted because of identifying information]*  Being with my kids makes me not want to smoke. Their health and my own makes me always reconsider my choices. | “We don’t care about our health, but we do care about the health of our animals.” |
| *Health consciousness:*  Pets or Animals (P) | 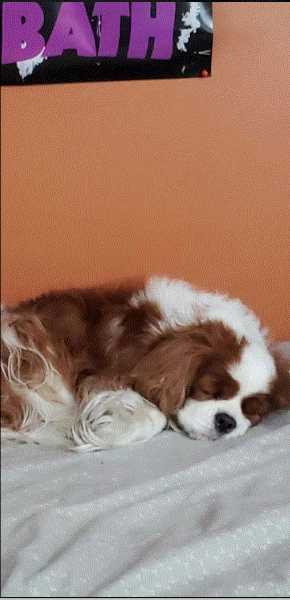  I know I'm late on this but my dog deff makes me wanna not smoke, since usually when I'm outside and would have a chance to smoke it's when I'm walking him | “because I remember reading a bunch of articles about how secondhand smoke affects pets. And I really love my cat and I don’t want him to get any ill effects from me smoking. So, whenever I do, I try to do it on the porch and I try to lock him out, which he hates but – this was a preventative factor also because he was sitting on my lap. And that’s a law that you can’t get up when your cat’s sitting on your lap.” |
|  |  |  |
| *Social influences:*  Friends or Peers (P,R) | 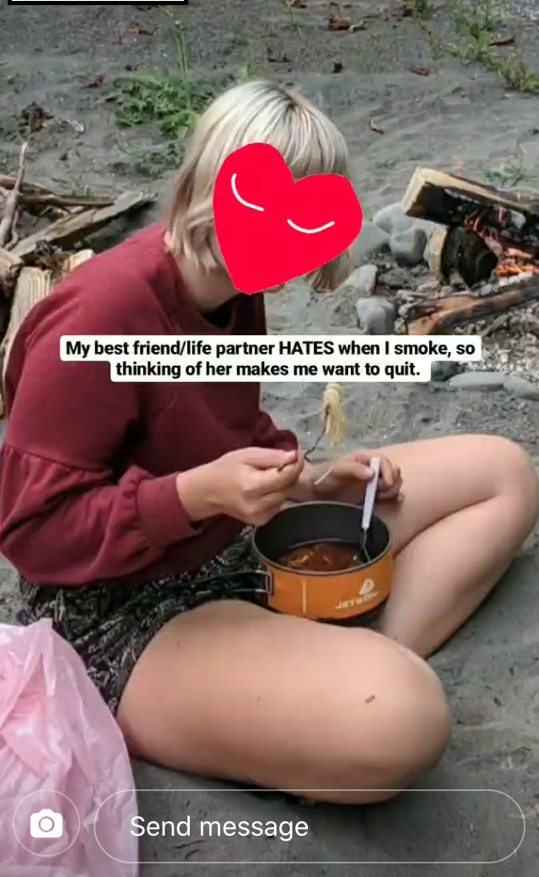 | Like I find it really hard to resist smoking if I see other people doing it, even though for me, smoking is almost entirely like a result of psychological [triggers]. I don’t really get much in the way of like physical cravings, or even like withdrawal symptoms if I don’t smoke. It’s just there are things that make me want to smoke, and seeing anyone else with a cigarette or hearing anybody else talk about having a cigarette is one of those things. (R) |
| *Social influences:*  Family Pressure (P,R) | 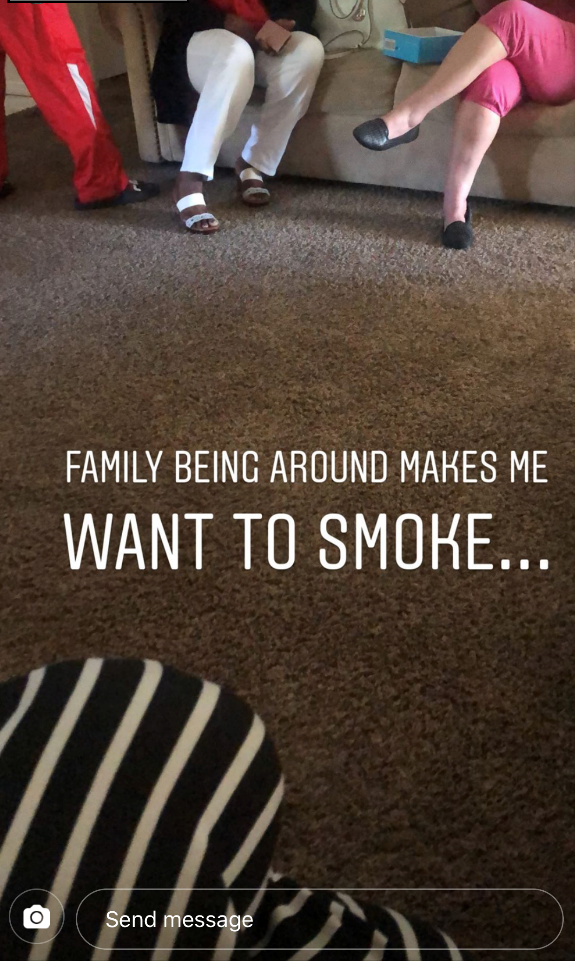 | “My family in particular, I feel like - I feel strongly compelled to continue keeping from them that I have been smoking, because my parents are both ex-smokers who both quit after a lot of hard work, and we were - me and my siblings were raised hearing never smoke, it’s not worth starting, and then you - and most people can’t stop. And, oh, look at me and your mom. We’re so lucky we quit. We could still - they would just be so disappointed.” (P) |
|  |  |  |
| *Routine behaviors:*  Commuting (R) | 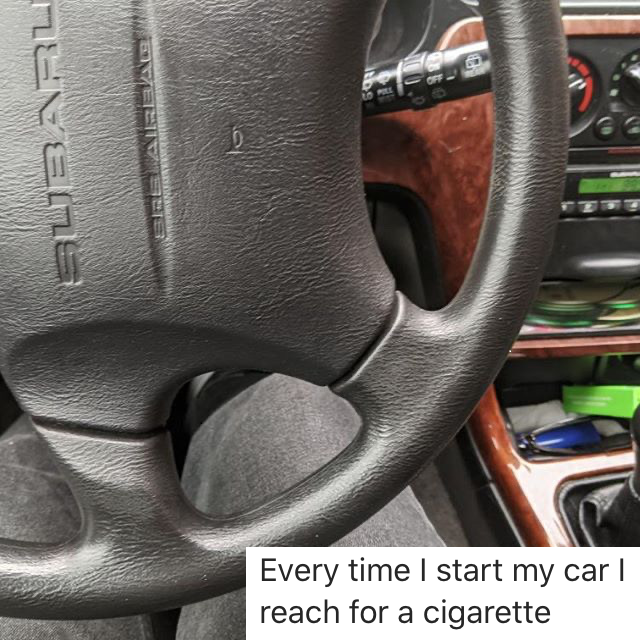 | “I think I was actually trying not to smoke that morning, and it was just such a habitual thing to smoke when I get in my car that the risk factor overcame my motivation not to smoke.” |
| *Routine behaviors:*  Taste of cigarettes (P, R) | 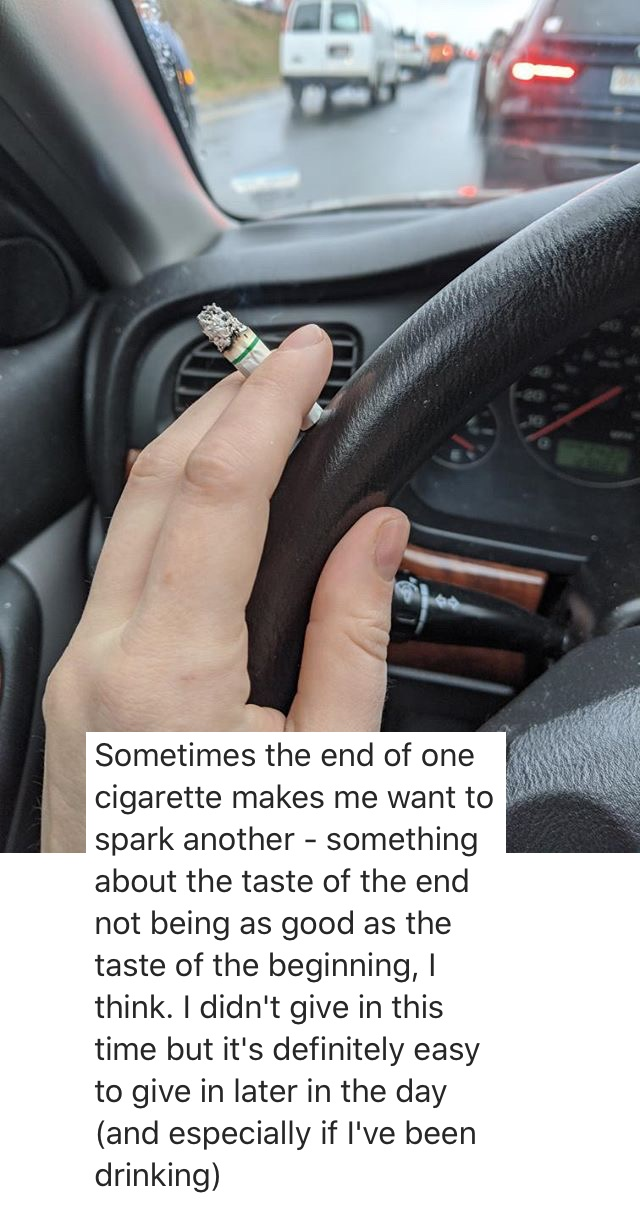 | “They were eight bucks, and they’re nasty. Made me not want to smoke, and I didn’t actually end up smoking that pack.” (P) |
|  |  |  |
| *Routine behaviors:*  Alcohol Use (R) | 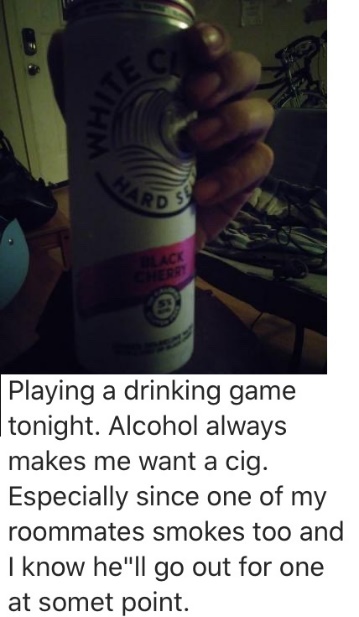 | “And also with drinking or other things, you reach a limit, right? So like I drink a lot, and then I’m super drunk and my friends are like, okay, like, maybe you shouldn’t have another glass. And it’s like but that’s because I’m like obliterated. But a cigarette, my friends don’t bat an eyelash when I smoke.” |
| *Routine behaviors:*  Distractions (P) | 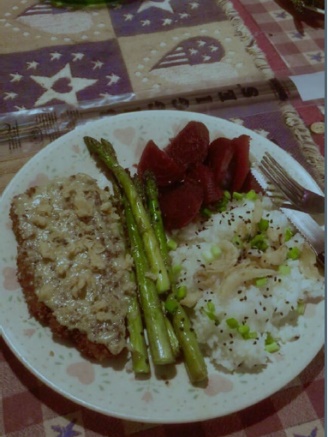  Cooking dinner tonight got me not to smoke for a good few hours haha | “But something that - I feel like distractions are the most direct one in my life of like - I was writing a paper earlier today for one of my classes that has been like occupying all my attention over the past few days. So even if I do have a craving to smoke, I don’t have time to. So that’s definitely a huge distraction that I guess is like a protective factor.” |
|  |  |  |
| *Environmental cues:*  Environmental/Situational (P,R) | 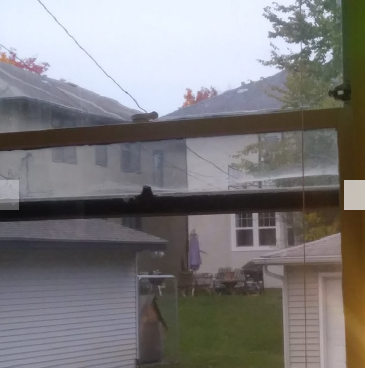  Cold, wet, boring, and grey day. Nice time for a cigarette (R) | “You’re like, I’m sick or the weather is s***, so I’m just – sometimes just kinda like, I can’t smoke because I can’t.” (P) |
| *Environmental cues:*  Public spaces (P) | 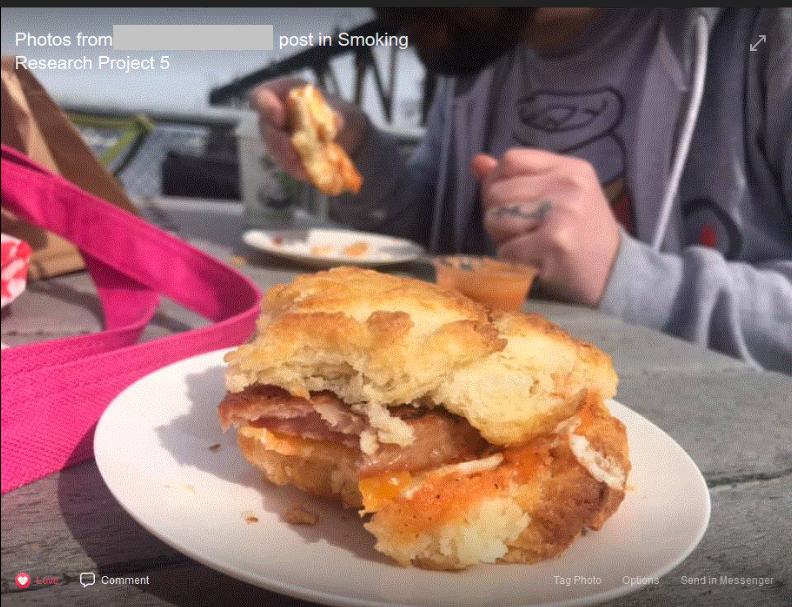  Today I craved a cigarette after enjoying this delightful biscuit sandwich. I love to have a smoke while walking around. but it was a public park so I waited until I got home | “…really good ducks, though, because this park doesn’t want the ducks to eat the cigarette butts. And I agree.” |
